# The Iowa University Again

**Published:** 1852-08

**Authors:** 


*The Iowa University again.*

In the July No. of the resuscitated *Western Medico-Chirurgical Journal*, we have an apology for a defence of the course of a certain Professor of the Iowa Medical School, in making charges which he could not substantiate, and pledges which he did not attempt to fulfil. We say an apology, for the professor in question fairly dodges the point in dispute and endeavors to escape by raising false issues

The first trick of this kind, is in showing in six pages of his Journal, that the former "College of Physicians and Surgeons of the Upper Mississippi," &c., is now actually the medical department of the Iowa University, and that, therefore, we uttered a falsehood in saying that he, the Professor, *assumed* to represent that University. Low as is our opinion of the literature of Keokuk, we cannot believe that sense and dictionaries are so scarce there that the definition of assume is totally unknown. It simply means to take upon oneself; it may be done wrongfully or rightfully; or, as in his case, arrogantly and without any authorization, as, by his own showing, he seems to have done. For it does not follow, that a teacher in one department is authorized to speak for and represent the whole University, and among the powers delegated this is not mentioned.

But we are not to be diverted from the point at issue by that device. Since the question of a reduction of fees has been brought out as a charge, and those who make it have skulked away to prevent its being met, we will put on record here the facts of the case, for the benefit of all such as may be interested in knowing them. They are as follows:

An agreement was entered into, dated Jan. 10, 1847, between the Indiana Medical College at La Porte and the Medical College at Chicago in regard to the fees, which contained the following clause:—"That no student be admitted to the lectures except upon the condition of paying at least one half of the lecture fees in advance, and giving a note for one year with approved security for the remainder."

It was provided that in case of violation of the agreement, it might be terminated by giving notice, and stipulated that any neglect to enforce the agreement or reduction of fees should be considered a violation. This agreement was faithfully adhered to by the Rush Medical College.

The Indiana Medical College immediately procured a bill to be passed, authorizing them to take two students from each county of the State at half-price, provided the county commissioners (officers who have charge of the paupers), should certify that they were poor, of good moral character, &c. This law was approved Feb. 16, 1848, just thirty-two days after the date of the agreement above quoted; it was accepted and acted upon by the Indiana College, and the certificate of poverty taken as cash, and credit given for the balance. The fees were at that time $65, one-half of which would be $32½.

It was only after the next course of lectures had commenced that the trick was discovered; notice of termination of the agreement was given, and the fees reduced to $35. In face of these facts it is, that the Iowa school, the legitimate successors of the Laporte school, and possessed as it seems of their archives, bring a charge against us of reducing the fees for the purpose of injuring them.

It should be added, that Indiana has 80 or 90 counties, two from each of which would about double the largest class *ever* assembled at Laporte.

That charge we have long been desirous of meeting in public, but no opportunity has been granted.

The evidence brought by the Iowa professor in support of his charge, is curious and characteristic. It is, that Dr. Meek, Dr. Herrick, one of their own students, and others, made offers to different members of their class of lower fees, if they would leave, &c. In reference to these charges, we have simply to say, that no such offers were ever made on the part of any authorized officer of the College.

To all the letters sent, and there were numbers, the uniform answer by the President or Secretary of the Faculty, was to inform them of the amount of our fees, and inform the applicant that no variation whatever would be made.

But a case is stated, in which one of the professors at Laporte, under an assumed name, by himself or another, wrote a letter, making false representations and asking for admission on lower terms. It was well known at the time that some such communication had been received by different members of the Faculty, and they were all answered officially; and if any one replied to them in the spirit in which they were written, for the purpose of mystifying that "fine little boy," and amusing himself, as "one of our boys" had done, the responsibility rests with him. No reply of the kind was written by any authorized person offering other than our published terms, and answers stating them were officially sent to every one of those letters.

The Professor states that he was prevented by a domestic affliction from attending the meeting at Richmond. This, for one individual, is a reason which is entitled to respect; but surely it need not have prevented him for a whole year from supporting his charge before the committee, or of having some other member of the university present to represent it. The excuse shows what we have already stated, that the attack was a personal matter. The medical department of the University, like its prototype, "the College of Physicians and Surgeons of the Upper Mississippi Valley," *et al* is in fact strictly a private speculation; if fees were high it might be made to pay, otherwise, it could not; and it was to be expected that the firm that owned it should be anxiou s t have the prices high.

The conclusion of the Professor's defense, like a lady's postscript, contains the gist of the whole matter.

"*They should re-organize their school upon just and honorable principles.*" *i. e.*, expel a couple of professors to make room for certain other persons, and "*determine the question of fees* as they may think proper."

Fairly given up the question of fees, it only remains for them to be consistent and reduce their own fees, which is evidently the next step to be expected from them. B.

